# Efficacy and Safety of Oral Supplementation with Liposomal Iron in Non-Dialysis Chronic Kidney Disease Patients with Iron Deficiency

**DOI:** 10.3390/nu16091255

**Published:** 2024-04-24

**Authors:** Davide Cesarano, Silvio Borrelli, Giorgia Campilongo, Annarita D’Ambra, Federica Papadia, Carlo Garofalo, Antonia De Marco, Federica Marzano, Chiara Ruotolo, Loreto Gesualdo, Pietro Cirillo, Roberto Minutolo

**Affiliations:** 1 Unit of Nephrology, Department of Advanced Medical and Surgery Sciences of University of Campania “Luigi Vanvitelli”, 80138 Napoli, Italy; davidecesarano1@gmail.com (D.C.); annaritadambra4@gmail.com (A.D.); carlo.garofalo@unicampania.it (C.G.); fedemarzy@hotmail.it (F.M.); chiara.ruotolo@yahoo.it (C.R.); roberto.minutolo@unicampania.it (R.M.); 2 Nephrology, Dialysis and Transplantation Unit, University of Bari “Aldo Moro”, 70124 Bari, Italy; campilongogio@gmail.com (G.C.); f.papadia7@gmail.com (F.P.); antonella.demarco16@gmail.com (A.D.M.); loretoge60@gmail.com (L.G.); cirillo.pietro@yahoo.it (P.C.)

**Keywords:** iron, liposomal iron, CKD, anemia, iron deficiency

## Abstract

Introduction. Iron deficiency is common in patients with non-dialysis-dependent chronic kidney disease (NDD-CKD). Oral iron supplementation is recommended in these patients, but it is associated with a higher incidence of gastrointestinal adverse reactions. Liposomal iron therapy has been proposed as a new iron formulation, improving iron bioavailability with less side effects; however, few data are available in patients with NDD-CKD. Methods. We designed a single-arm pilot study to evaluate the efficacy of liposomal iron administered for six months in correcting iron deficiency (defined as serum ferritin < 100 ng/mL and/or transferrin saturation < 20%) in patients with NDD-CKD stages 1–5. The primary endpoints were the achievement of serum ferritin ≥ 100 ng/mL and transferrin saturation ≥ 20%. Secondary outcomes were hemoglobin (Hb) changes and the safety of liposomal iron. Results. The efficacy population included 34/38 patients, who completed at least one visit after baseline. Liposomal iron increased the achievement of transferrin saturation targets from 11.8% at baseline to 50.0% at month 6 (*p* = 0.002), while no significant correction of serum ferritin (*p* = 0.214) and Hb was found (*p* = 0.465). When patients were stratified by anemia (Hb < 12 g/dL in women and Hb < 13 g/dL in men), a significant improvement of transferrin saturation was observed only in anemic patients (from 13.3 ± 5.8% to 20.2 ± 8.1%, *p* = 0.012). Hb values slightly increased at month 6 only in anemic patients (+0.60 g/dL, 95%CI −0.27 to +1.48), but not in those without anemia (+0.08 g/dL, 95%CI −0.73 to +0.88). In patients taking at least one dose of liposomal iron (safety population, *n* = 38), the study drug was discontinued in eight patients due to death (*n* = 2), a switch to intravenous iron (*n* = 2), and the occurrence of side effects (*n* = 4). Conclusions. The use of liposomal iron in patients with NDD-CKD is associated with a partial correction of transferrin saturation, with no significant effect on iron storage and Hb levels.

## 1. Introduction

Iron deficiency is prevalent in patients with non-dialysis-dependent chronic kidney disease (NDD-CKD) and represents the most common and reversible cause of anemia and resistance to erythropoiesis-stimulating agents in these patients [1]. Iron deficiency is due to either the depletion of iron stores (absolute iron deficiency) or the impaired body’s ability to utilize the iron accumulated in the tissues (functional iron deficiency), which results from the mismatch between supply and demand or reticuloendothelial cell iron blockade. These forms of iron deficiency frequently occur in non-dialysis populations [2,3]. Absolute iron deficiency is due to the severe reduction in iron stores in the liver and reticular-endothelial system and is commonly diagnosed by a reduction in serum ferritin with or without low transferrin saturation. Functional iron deficiency is characterized by a reduction in transferrin saturation associated with replenished iron deposits (normal or increased serum ferritin), which are not immediately available for erythropoiesis. Functional iron deficiency is primarily related to inflammatory status and the consequent increased hepcidin levels. Indeed, inflammatory cytokines (mainly interleukin 6) increases serum hepcidin release by hepatocytes, which in turn inhibits ferroportin expression, a protein playing a crucial role in the iron efflux from enterocytes and macrophages into the bloodstream. Under these conditions, the iron available for erythropoiesis is low, despite the presence of full iron stores [3].

Oral iron supplementation is recommended as first-line treatment for iron deficiency correction in NDD-CKD [4,5,6] before considering the intravenous route because, despite being more effective than the oral route, it is associated with a higher risk of hypersensitivity reactions and higher costs [7,8]. Indeed, intravenous iron may potentially increase the risk of iron overload [9] and the incidence of infections, at least for patients with a central venous catheter [10]. Moreover, intravenous administration is logistically complex and is associated with adverse events regarding the site of injection [7,8].

Notably, the bioavailability of oral iron salts is low, and when administered at a high dose it can further impair absorption by up-regulating hepcidin levels [11]. More importantly, the efficacy of oral iron supplementation is frequently impaired by a higher incidence of adverse gastrointestinal side effects occurring in 13–20% of patients, which can reduce treatment compliance [1,12]. Finally, oral iron absorption can also be reduced when co-administered with food or certain medications affecting gastric pH (e.g., antacids and proton pump inhibitors) [2,3,12].

Liposomal iron is a specialized delivery system in which 15 to 30 mg of inorganic iron (according to different formulations) is encapsulated within tiny liposomes. These liposomes act as protective shells, enhancing the absorption of iron in the gastrointestinal tract and ensuring efficient transport into the bloodstream, resulting in optimized bioavailability. Furthermore, liposomal iron is associated with a lower incidence of adverse gastrointestinal effects, thus increasing compliance to the iron supplementation [13].

Liposomal iron therapy has been proposed as a new iron formulation, improving iron bioavailability with less side effects; however, few data are available in patients with NDD-CKD. Therefore, we carried out an observational study to evaluate the effect associated with liposomal iron use in patients with NDD-CKD affected by either functional or absolute iron deficiency.

## 2. Methods

This is a single-arm pilot study designed to evaluate the efficacy of liposomal iron in correcting iron deficiency in patients with NDD-CKD stages 1–5 who were consecutively seen in two nephrology units from 1 June 2022 to 1 January 2023.

Inclusion criteria were (a) the presence of iron deficiency, defined as serum ferritin < 100 ng/mL (absolute iron deficiency) and/or transferrin saturation < 20% (relative iron deficiency) and (b) the diagnosis of NDD-CKD, defined as GFR < 60 mL/min/1.73 m^2^ or GFR > 60 mL/min/1.73 m^2^ and albuminuria > 30 mg/day. Patients under kidney replacement therapy or with acute hemorrhages, chronic inflammatory bowel disease, liver or kidney transplantation, immunosuppressive therapy, advanced liver or heart failure, or active neoplasms were excluded Treatment with intravenous iron in the previous six months and other causes of anemia (e.g., thalassemia, myelofibrosis, etc.) were also considered exclusion criteria. All participating patients signed an informed consent form. The study was conducted according to the guidelines of the Declaration of Helsinki and approved by the Institutional Review Board as part of a previous data-banking project (approval number n. 0013983/i of 5 May 2022 of University of Campania Luigi Vanvitelli, Naples, Italy).

### 2.1. Data Collection

At baseline, demographic data (age, gender), anthropometric measures (height, body weight, body mass index), and clinical history were collected. In addition, at each visit the following lab tests were assessed: serum creatinine, serum parathyroid hormone, red blood cell count, hemoglobin, serum C-reactive protein (CRP), serum phosphate, serum iron, serum transferrin, and serum ferritin. Transferrin saturation was calculated according to the following formula: (serum iron/serum transferrin) × 70.9 and expressed as a percentage. Albuminuria was assessed as 24 h urinary albumin excretion. GFR was estimated using the CKD Epidemiology Collaboration equation [14].

Anemia was defined as either Hb < 12 g/dL in women and <13 g/dL in men in patients not receiving erythropoietin-stimulating agents or in the presence of treatment with erythropoietin-stimulating agents independently from Hb levels, according to the current guidelines [4,5,6,15]

### 2.2. Study Design and Endpoints

The study lasted 6 months with pre-specified timing collection (baseline, month 1, month 3, and month 6) to assess the efficacy of liposomal iron in correcting iron deficiency. Each pill of liposomal iron contained 30 mg of elemental iron and 19 mg of Vitamin C. All patients received liposomal iron for six months according to the following schedule: 30 mg/day twice daily for the first month, followed by 30 mg daily for the remaining five months. Adherence to the iron supplementation was assessed in patients completing the study by counting the pills returned by patients at each visit. Adherence was calculated in patients performing all the pre-specified study visits; this parameter was used as a measure of the compliance to the prescription in the absence of specific causes (side effects) leading to the drug discontinuation.

Primary endpoints were changes from the baseline of either serum ferritin or transferrin saturation after six months of iron supplementation. Secondary outcomes measured at the month-6 visit were (a) the achievement of the target for serum ferritin (≥100 ng/mL) and transferrin saturation (≥20%), (b) the hemoglobin changes from baseline, (c) the cumulative incidence of the side effects associated with liposomal iron, and (d) the need for other iron supplementation (different oral iron compounds or intravenous iron).

### 2.3. Statistical Analysis

Continuous variables were reported as either mean ± standard deviation (SD), mean and 95%CI, or median and interquartile range (IQR) according to the distribution of each variable. Categorical variables were reported as percentages. Changes in continuous variables during the study were analyzed by linear mixed models, assuming an unstructured covariance matrix; this was done to consider the correlation between repeated measures and missing points for patients not completing the study. Changes from baseline in the prevalence of corrected serum ferritin and transferrin saturation values were evaluated at months 1, 3, and 6 using the McNemar test. Data were analyzed using SPSS statistics version 26 (IBM, Armonk, NY, USA).

## 3. Results

Thirty-eight patients, who received at least one dose of liposomal iron, were enrolled in the study (safety population). The efficacy population included 34/38 patients with at least one control visit after baseline (four patients dropped out in the first month for the following causes: two for diarrhea, one for dyspepsia and constipation, and one for death).

The main clinical data of the safety population at baseline are reported in Table 1. Briefly, patients were, on average, 65.3 years old, 47% were women (all women were in menopause), and 50% showed GFR < 30 mL/min/1.73 m^2^. Clinically relevant inflammation and hyperparathyroidism were not present in the large majority of our patients (Table 1). Indeed, CRP > 10 mg/dL was detected in four patients at baseline but remained above this threshold in only one subject during the study; PTH level above 300 pg/mL was detected in one patient only at baseline visit. The clinical characteristics of patients enrolled in the two clinics did not differ.

In the efficacy population, anemia was detected in 22/34 patients (67.7%) at baseline; in particular, 5 received erythropoiesis stimulating agents (all but one had Hb < 12 g/dL) and 17 had hemoglobin below the sex-specific cut-off without receiving erythropoiesis stimulating agents. Baseline serum ferritin was 44 (IQR 19–82) ng/mL and transferrin saturation was 14.3 ± 5.9%, corresponding to a prevalence of serum ferritin <100 ng/mL of 78.9%, and transferrin saturation <20% of 89.5%. Prevalence of serum ferritin and TSAT below the thresholds were detected in 64.7% of patients.

**Figure 1:**
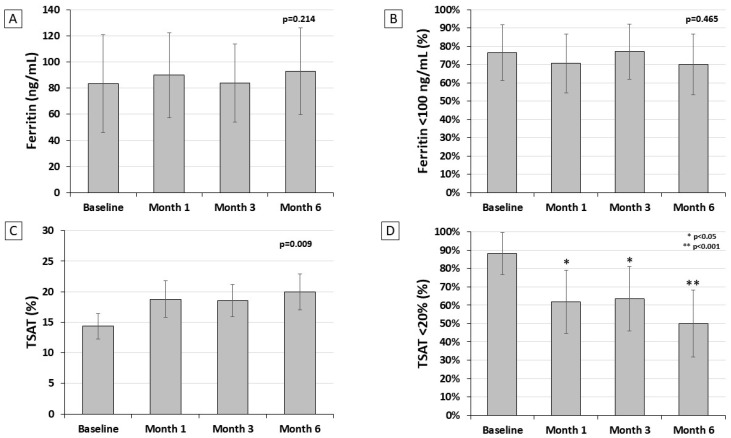
Mean levels of serum ferritin (panel (**A**)) and transferrin saturation (TSAT) (panel (**C**)) and prevalence of low ferritin (panel (**B**)) and low transferrin saturation (TSAT) (panel (**D**)) during the study in the efficacy population (*n* = 34).

Liposomal iron induced a progressive increase in transferrin saturation from 14.3 ± 5.9% at baseline to 20.0 ± 7.6% at month 6 (*p* = 0.009, Figure 1C), whereas no difference was detected for serum ferritin (*p* = 0.214, Figure 1A). Accordingly, at the end of the study the prevalence of transferrin saturation in target (>20%) significantly increased from 11.8% to 50.0% (*p* = 0.002, Figure 1D), while the prevalence of serum ferritin target (>100 ng/mL) was slightly and not significantly improved (from 23.5% to 30.0%, *p* = 0.465, Figure 1B).

**Figure 2:**
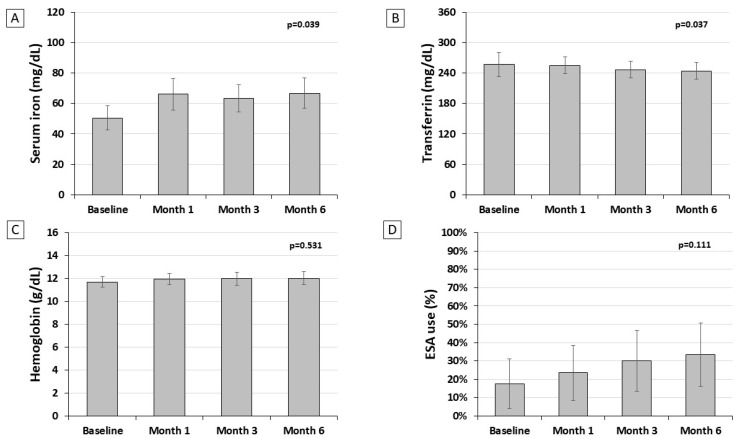
Mean serum iron (panel (**A**), serum transferrin (panel (**B**)), hemoglobin (panel (**C**)), and prevalence of ESA use (panel (**D**)) during the study in the efficacy population (*n* = 34). Data are mean values and error bars are 95% CI.

Figure 2 shows the mean serum levels of the main parameters assessed to evaluate iron metabolism. We found a significant trend toward an increase in serum iron and a reduction in serum transferrin during the six months of therapy; conversely, liposomal iron supplementation did not significantly change the hemoglobin level, and nor did the use of erythropoiesis-stimulating agents. Among the treated patients, the dose of erythropoiesis-stimulating agents (6000 IU/week, IQR 2800–8000) remained unchanged during the study (*p* = 0.329).

When patients were stratified by anemic status (Table 2), a significant improvement of TSAT was observed in patients with anemia (from 13.3 ± 5.8% to 20.2 ± 8.1%, *p* = 0.012), whereas no significant improvement was found in patients without anemia (*p* = 0.093). A slight (but not statistically significant) improvement of serum ferritin and transferrin saturation was observed in non-anemic patients.

Hemoglobin values did not significantly increase at the month-6 visit in either anemic (+0.60 g/dL, 95%CI −0.27 to +1.48) or non-anemic (+0.08 g/dL, 95%CI −0.73 to +0.88) patients. During the study, the proportion of patients with an increase in hemoglobin >1 g/dL did not differ between patients with and without anemia (36.4% and 25.0%, respectively, *p* = 0.498).

Anemia is defined as hemoglobin < 12 g/dL in women and <13 g/dL in men. Data are mean (95% CI).

**Figure 3:**
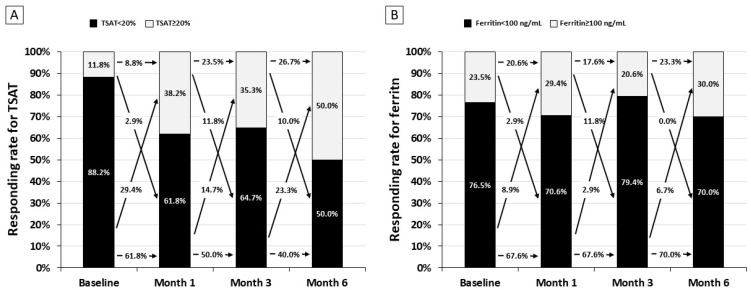
Responders to liposomal iron on transferrin saturation (TSAT, (**A**) and ferritin (**B**) at each visit.

As descriptive analysis, we evaluated the frequency of responders (Figure 3). After the first month of the therapy, controlled transferrin saturation (>20%) improved (from 11.8% to 38.2%), remained stable at month 3 (35.3%), and further increased at the following visit to 50.0% (Figure 3A). Conversely, the response rate for serum ferritin remained stable over 6 months (Figure 3B). The cumulative dose of elemental iron in these patients was 6244 ± 128 mg/patient.

In the safety population (*n* = 38), the study drug was discontinued in eight patients due to death (*n* = 2), a switch to intravenous iron (*n* = 2), and occurrence of side effects (*n* = 4) (Table 3). During the study, the four patients who discontinued the study drug reported nine side effects; these occurred predominantly after baseline (*n* = 7 in three patients) while two side effects (in one patient) were reported at the month-6 visit. We assessed the adherence to liposomal iron supplementation by evaluating the pill count in patients completing the study (*n* = 30). We found that adherence to treatment was 95.6%.

## 4. Discussion

The present pilot study has been designed to evaluate the potential benefit of liposomal iron in correcting iron deficiency in patients with NDD-CKD with or without anemia. We found that iron supplementation with liposomal compounds for six months was associated with a better achievement of the recommended goal for transferrin saturation (from 11.8 to 50.0%, *p* = 0.002), with a limited effect on the achievement of ferritin target (from 23.5% to 35%, *p* = 0.465). The impact on transferrin saturation was more pronounced in the subgroup of NDD-CKD patients with anemia, likely because the higher iron demand for stimulating erythropoiesis prevails over the need of replenishing iron stores. However, no significant effect was observed on either hemoglobin levels or the need for erythropoiesis-stimulating agents. We found that liposomal iron therapy was associated with a significant increase in serum iron (Figure 2A) and a reduction in serum transferrin (Figure 2B), whereas the level of serum ferritin did not change over time (Figure 1A). This effect translated into a significant improvement in transferrin saturation (Figure 1C), with a partial improvement in iron availability (Figure 1D), but not in the replenishment of stores (Figure 1C). The increased amount of iron carried to the bone marrow induced a slight but not significant increase in Hb levels (+0.32 g/dL) (Figure 2C). Therefore, it is conceivable that in patients affected by NDD-CKD, liposomal iron improves iron bioavailability, generating an iron influx into the bloodstream, which is quickly available for erythropoiesis. However, it is not enough to restore iron deposits, as confirmed by the persistence of low ferritin levels. It is important to underline that the severity of anemic status at baseline affects the efficacy of liposomal iron. Therefore, our findings suggest that a daily dose should be increased in order to provide a clinically relevant effect on anemia.

Liposomal iron is a sophisticated delivery system consisting of micronized particles of ferric sulfate enclosed in a phospholipid vesicle, which facilitates iron transport through the membrane cell [16]. These lipid structures work as iron carriers to prevent early degradation and inactivation in the gastric environment, making iron quickly available in the bloodstream. Because of these characteristics, liposomal iron is a therapeutic option to correct iron deficiency, particularly indicated for NDD-CKD patients, as it can potentially overcome the adverse effects on iron metabolism induced by an eventual increase in hepcidin levels with consequent lower expression of ferroportin on the basal membrane of enterocytes [3,11]. In this regard, it has been demonstrated by experimental studies in anemic mice that liposomal iron, in comparison with ferrous sulfate, did not induce an increase in hepcidin levels [17]; moreover, liposomal iron, as compared with ferrous sulphate, was associated with a beneficial effect on the intestinal microbiome [18].

Other studies with liposomal iron in NDD-CKD patients are reported in Table 4. In a prospective observational study from Spain, 37 patients with NDD-CKD stage 3 (mean GFR 42 mL/min/1.73 m^2^) and intolerance to other oral iron formulations received liposomal iron for twelve months. The authors found that, after six months of treatment with liposomal iron, hemoglobin increased by 0.49 mg/dL on average, while transferrin saturation and ferritin were not significantly modified [19]. However, no data were reported on the prevalence at baseline of iron deficiency and treatment with erythropoiesis-stimulating agents as well. Similarly, an Italian trial in NDD-CKD patient with iron-deficient anemia compared liposomal iron with intravenous iron therapy [20]. The authors reported a significant increase in hemoglobin of 0.6 g/dL on average after three months of treatment with liposomal iron. At variance with our findings, Pisani et al. reported that transferrin saturation did not increase from baseline; conversely, the lack of change in ferritin levels was in agreement with our results [20]. Finally, a recent trial compared the effects of three different oral iron formulations on anemia correction in 62 patients with iron-deficient anemia not on dialysis (GFR 30–60 mL^7^ min/1.73 m^2^) [21]. Patients were randomized to three treatment groups for six months: 105 mg/day of ferrous sulfate (Group 1; N = 20), 30 mg of ferric sodium EDTA (Group 2; N = 22), and 30 mg of ferric liposomal formulation (Group 3; N = 20). The authors reported that hemoglobin levels and iron parameters improved with liposomal iron and ferric sodium EDTA, but not with ferrous sulfate [20]. These findings are hardly comparable to our results because Giliberti et al. enrolled only patients with functional iron deficiency (low transferrin saturation but normal ferritin levels) without any treatment with erythropoiesis-stimulating agents. Therefore, it is likely that the iron needs in those subjects were completely different from our patients.

Oral iron supplementation is frequently associated with a higher risk of gastrointestinal adverse events [3]. Enteric iron is present in the trivalent form (Fe^3+^) and needs to be reduced to the ferrous form (Fe^2+^) by duodenal cytochrome B reductase (D-cyt-B). Indeed, only divalent iron can be transported from the intestinal lumen into the enterocyte through a specific carrier (transporter protein Divalent Metal Transporter 1, DMT-1), located on the apical surface of enterocytes. However, exposure to Fe^3+^ overload, which is not absorbable, can damage the intestinal barrier by inducing local inflammation (e.g., iron-induced reactive oxygen species) with a substantial impact on gut microbial communities [18]. Moreover, iron overload can lead to ferroptosis, a form of programmed cell death mediated by iron-dependent lipid peroxidation within cell membranes [2]. Coating iron with liposomal external shell limits the damage on intestinal cells because it prevents the interaction between iron and the intestinal mucosa, thus minimizing gastrointestinal side effects [13,16]. This is a critical point as the onset of gastrointestinal adverse reactions (e.g., diarrhea, constipation, and dyspepsia) remarkably affects adherence to oral iron therapy. We found a prevalence of gastrointestinal effects during the six months of liposomal iron therapy (10%) lower than that currently reported in the literature [1,12]. At the same time, among patients completing the study (N = 30), we found a good compliance with iron therapy, as testified by a very high adherence to the treatment (96%). This result is in line with other experiences reporting a very high compliance (Table 4) [19,20,21].

Liposome technology seems effective in protecting intestinal cells from iron toxicity from the actual iron content in each pill (30 mg). However, our findings raise a possible question about the dose of iron enclosed in the liposome. Indeed, as observed in Figure 3, a significant part of the effect on transferrin saturation and serum ferritin was seen in the first month when the dose of iron liposome was administered at higher dose (60 mg/daily) as compared with the dose used in the following five months (30 mg/day). This finding suggests that, for supporting the increased iron demand in patients with NDD-CKD, they should be treated with a higher dose of liposomal iron. Future studies designed with high doses of liposome iron in each pill (in order to limit the pill burden) would be desirable to test the hypothesis that liposomal iron at higher doses may improve efficacy by confirming at the same time the good safety profile of this new iron formulation.

Our study has limitations inherent to the study design and the small sample size and the lack of a control group, which prevents any causative conclusion. However, this pilot study aims to provide some insights into the use of this iron compounds in order to have sufficient data to better design future studies in patients with NDD-CKD.

## 5. Conclusions

In conclusion, in patients with NDD-CKD, oral supplementation with liposomal iron allows a partial correction of transferrin saturation in half of patients treated for six months, with no significant effect on serum ferritin and hemoglobin levels. The greater effect detected in the first month associated with doubling liposomal iron dose suggests that a more aggressive dosing strategy is required in patients with NDD-CKD to achieve the full correction of iron-deficient anemia.

## Figures and Tables

**Table 1:** Demographic and clinical characteristics of patients at baseline.

	Safety Population (*n* = 38)
Age (years)	65.3 ± 15.2
Men (%)	52.6
Body mass index (kg/m^2^)	26.5 ± 4.2
Diabetes mellitus (%)	35.1
eGFR (mL/min/1.73 m^2^)	38.5 ± 28.8
eGFR > 60 (%)	15.8
eGFR 60–30 (%)	34.2
eGFR < 30 (%)	50.0
Proteinuria (g/day)	0.24 (0.07–1.13)
Serum phosphate (mg/dL)	3.80 ± 0.78
Parathyroid hormone (pg/mL)	102 (65–158)
C-reactive protein (mg/L)	0.08 (0.06–1.60)
Hemoglobin (g/dL)	11.6 ± 1.3
Hemoglobin < 12 (F) or <13 g/dL (M) (%)	65.8
Hemoglobin < 11 g/dL (%)	26.3
Transferrin saturation (%)	14.3 ± 5.8
Transferrin saturation < 20% (%)	89.5
Serum ferritin (ng/mL)	44 (19–82)
Serum ferritin < 100 ng/mL (%)	78.9
Serum iron (µg/dL)	51 ± 22
Transferrin (mg/dL)	261 ± 65
Erythropoiesis stimulating agents use (%)	21.1
Systolic/diastolic blood pressure (mmHg)	136 ± 15/77 ± 9

Data are mean ± SD, percent or median (IQR). eGFR, estimated glomerular filtration rate.

**Table 2:** Serum levels of ferritin and hemoglobin and transferrin saturation (%) at baseline and at each point during the treatment with liposomal iron in the efficacy population stratified by anemia status.

	Baseline	Month 1	Month 3	Month 6	*p*
Without anemia (*n* = 12)					
Ferritin (ng/mL)	44 (57–72)	63 (29–99)	64 (24–104)	73 (26–120)	0.092
Transferrin saturation (%)	16.3 (12.4–20.1)	20.1 (15.4–24.7)	19.1 (13.9–24.3)	20.5 (14.5–26.5)	0.093
Hemoglobin (g/dL)	13.0 (12.5–13.6)	12.9 (12.2–13.7)	13.3 (12.7–13.9)	13.1 (12.1–14.0)	0.480
With anemia (*n* = 22)					
Ferritin (ng/mL)	105 (45–160)	105 (58–151)	94 (52–137)	103 (57–150)	0.394
Transferrin saturation (%)	13.3 (10.8–15.9)	18.1 (13.9–22.20	18.2 (14.9–21.4)	20.2 (16.1–24.4)	0.012
Hemoglobin (g/dL)	11.0 (10.6–11.3)	11.4 (10.9–12.0)	11.3 (10.7–12.0)	11.6 (11.0–12.1)	0.268

**Table 3:** Side effects in iron-deficient CKD patients treated with liposomal iron.

	Safety Population (*n* = 38)
Death	2 (5.3%)
Switch to IV iron for inefficacy	2 (5.3%)
Side effects	4 (10.5%)
Abdominal pain	4 (10.5%)
Nausea	2 (5.3%)
Diarrhea	2 (5.3%)
Constipation	1 (2.6%)

**Table 4:** Effects of liposomal iron supplementation in NDD-CKD patients in previous studies in comparison with our results.

Author (Year)	Main Inclusion Criteria	Baseline Values	Month-6 Values #	Adherence
Pisani (2015) [20]	Hb ≤ 12 g/dL, TSAT ≤ 25%, and Ferritin ≤ 100 ng/mL, ±ESA	Hb 10.8 ± 0.6 g/dL TSAT 16.5 ± 2.2% Ferritin 71 ± 24 ng/mL	Hb 11.4 ± 0.8 * g/dL TSAT 18.3 ± 4.3% Ferritin 86 ± 31 ng/mL	95.8%
Montagud-Marrah (2020) [19]	Intolerance to oral iron, ESA users not reported	Hb 12.0 ± 1.4 g/dL TSAT 16.8 ± 6.2% Ferritin 91 ± 104 ng/mL	Hb 12.5 ± 1.6 * g/dL TSAT 19.4 ± 7.9% Ferritin 96 ± 87 ng/mL	100%
Giliberti (2022) [21]	Hb 10–12 (women) 10–13 g/dL (men), TSAT < 20% and ferritin > 100 ng/mL, no ESA	Hb 10.6 ± 0.14 g/dL TSAT 17.4 ± 4.3% Ferritin 269 ± 57 ng/mL	Hb 10.8 ± 0.2 * g/dL TSAT 19.6 ± 2.7 *% Ferritin 218 ± 46 * ng/mL	100%
Present study	Any Hb, TSAT < 20% and/or ferritin < 100 ng/mL, ±ESA	Hb 11.7 ± 1.3 g/dL TSAT 14.4 ± 6.0% ± Ferritin 83 ± 107 ng/mL	Hb 12.0 ± 1.5 g/dL TSAT 20.0 ± 7.8 *% Ferritin 93 ± 94 ng/mL	95.6%

# The study by Pisani et al. lasted 3 months. Hb, hemoglobin; TSAT, transferrin saturation; ESA, erythropoiesis-stimulating agents. * *p* < 0.05.

## Data Availability

Data are contained within the article.
